# Supplementary material for: OpenMM 7: Rapid development of high performance algorithms for molecular dynamics
Source: PLoS Comput Biol. 2017 Jul 26;13(7):e1005659. doi: 10.1371/journal.pcbi.1005659 (PMC5549999; doi:10.1371/journal.pcbi.1005659)
Supplement: S1 Source Code — (ZIP) [file pcbi.1005659.s002.zip › OpenMM-7.0.1-Source/docs/api-python/generated/simtk.openmm.mtsintegrator.MTSIntegrator.html]

MTSIntegrator — OpenMM 7.0.1.dev-5e86c4f documentation


# MTSIntegrator¶

*class* `simtk.openmm.mtsintegrator.``MTSIntegrator`(*dt*, *groups*)¶
:   MTSIntegrator implements the rRESPA multiple time step integration algorithm.

    This integrator allows different forces to be evaluated at different frequencies,
    for example to evaluate the expensive, slowly changing forces less frequently than
    the inexpensive, quickly changing forces.

    To use it, you must first divide your forces into two or more groups (by calling
    setForceGroup() on them) that should be evaluated at different frequencies. When
    you create the integrator, you provide a tuple for each group specifying the index
    of the force group and the frequency (as a fraction of the outermost time step) at
    which to evaluate it. For example:

    ```
    integrator = MTSIntegrator(4*femtoseconds, [(0,1), (1,2), (2,8)])
    ```

    This specifies that the outermost time step is 4 fs, so each step of the integrator
    will advance time by that much. It also says that force group 0 should be evaluated
    once per time step, force group 1 should be evaluated twice per time step (every 2 fs),
    and force group 2 should be evaluated eight times per time step (every 0.5 fs).

    A common use of this algorithm is to evaluate reciprocal space nonbonded interactions
    less often than the bonded and direct space nonbonded interactions. The following
    example looks up the NonbondedForce, sets the reciprocal space interactions to their
    own force group, and then creates an integrator that evaluates them once every 4 fs,
    but all other interactions every 2 fs:

    ```
    nonbonded = [f for f in system.getForces() if isinstance(f, NonbondedForce)][0]
    nonbonded.setReciprocalSpaceForceGroup(1)
    integrator = MTSIntegrator(4*femtoseconds, [(1,1), (0,2)])
    ```

    For details, see Tuckerman et al., J. Chem. Phys. 97(3) pp. 1990-2001 (1992).

    `__init__`(*dt*, *groups*)¶
    :   Create an MTSIntegrator.

        |  |  |
        | --- | --- |
        | Parameters: | - **dt** (*time*) – The largest (outermost) integration time step to use - **groups** (*list*) – A list of tuples defining the force groups. The first element of   each tuple is the force group index, and the second element is the   number of times that force group should be evaluated in one time step. |

    Methods

    |  |  |
    | --- | --- |
    | `__init__`(dt, groups) | Create an MTSIntegrator. |

    Attributes

    |  |  |
    | --- | --- |
    | `BlockEnd` |  |
    | `ComputeGlobal` |  |
    | `ComputePerDof` |  |
    | `ComputeSum` |  |
    | `ConstrainPositions` |  |
    | `ConstrainVelocities` |  |
    | `IfBlockStart` |  |
    | `UpdateContextState` |  |
    | `WhileBlockStart` |  |

    `__delattr__`¶
    :   x.\_\_delattr\_\_(‘name’) <==> del x.name

    `__format__`()¶
    :   default object formatter

    `__getattribute__`¶
    :   x.\_\_getattribute\_\_(‘name’) <==> x.name

    `__hash__`¶

    `__reduce__`()¶
    :   helper for pickle

    `__reduce_ex__`()¶
    :   helper for pickle

    `__sizeof__`() → int¶
    :   size of object in memory, in bytes

    `__str__`¶

    `addComputeGlobal`(*self*, *variable*, *expression*) → int¶
    :   Add a step to the integration algorithm that computes a global value.

        |  |  |
        | --- | --- |
        | Parameters: | - **variable** (*string*) – the global variable to store the computed value into - **expression** (*string*) – a mathematical expression involving only global variables. In each integration step, its value is computed and stored into the specified variable. |
        | Returns: | the index of the step that was added |
        | Return type: | int |

    `addComputePerDof`(*self*, *variable*, *expression*) → int¶
    :   Add a step to the integration algorithm that computes a per-DOF value.

        |  |  |
        | --- | --- |
        | Parameters: | - **variable** (*string*) – the per-DOF variable to store the computed value into - **expression** (*string*) – a mathematical expression involving both global and per-DOF variables. In each integration step, its value is computed for every degree of freedom and stored into the specified variable. |
        | Returns: | the index of the step that was added |
        | Return type: | int |

    `addComputeSum`(*self*, *variable*, *expression*) → int¶
    :   Add a step to the integration algorithm that computes a sum over degrees of freedom.

        |  |  |
        | --- | --- |
        | Parameters: | - **variable** (*string*) – the global variable to store the computed value into - **expression** (*string*) – a mathematical expression involving both global and per-DOF variables. In each integration step, its value is computed for every degree of freedom. Those values are then added together, and the sum is stored in the specified variable. |
        | Returns: | the index of the step that was added |
        | Return type: | int |

    `addConstrainPositions`(*self*) → int¶
    :   Add a step to the integration algorithm that updates particle positions so all constraints are satisfied.

        |  |  |
        | --- | --- |
        | Returns: | the index of the step that was added |
        | Return type: | int |

    `addConstrainVelocities`(*self*) → int¶
    :   Add a step to the integration algorithm that updates particle velocities so the net velocity along all constraints is 0.

        |  |  |
        | --- | --- |
        | Returns: | the index of the step that was added |
        | Return type: | int |

    `addGlobalVariable`(*self*, *name*, *initialValue*) → int¶
    :   Define a new global variable.

        |  |  |
        | --- | --- |
        | Parameters: | - **name** (*string*) – the name of the variable - **initialValue** (*double*) – the variable will initially be set to this value |
        | Returns: | the index of the variable that was added |
        | Return type: | int |

    `addPerDofVariable`(*self*, *name*, *initialValue*) → int¶
    :   Define a new per-DOF variable.

        |  |  |
        | --- | --- |
        | Parameters: | - **name** (*string*) – the name of the variable - **initialValue** (*double*) – the variable will initially be set to this value for all degrees of freedom |
        | Returns: | the index of the variable that was added |
        | Return type: | int |

    `addUpdateContextState`(*self*) → int¶
    :   Add a step to the integration algorithm that allows Forces to update the context state.

        |  |  |
        | --- | --- |
        | Returns: | the index of the step that was added |
        | Return type: | int |

    `beginIfBlock`(*self*, *condition*) → int¶
    :   Add a step which begins a new “if” block.

        |  |  |
        | --- | --- |
        | Parameters: | **condition** (*string*) – a mathematical expression involving a comparison operator and global variables. All steps between this one and the end of the block are executed only if the condition is true. |
        | Returns: | the index of the step that was added |
        | Return type: | int |

    `beginWhileBlock`(*self*, *condition*) → int¶
    :   Add a step which begins a new “while” block.

        |  |  |
        | --- | --- |
        | Parameters: | **condition** (*string*) – a mathematical expression involving a comparison operator and global variables. All steps between this one and the end of the block are executed repeatedly as long as the condition remains true. |
        | Returns: | the index of the step that was added |
        | Return type: | int |

    `endBlock`(*self*) → int¶
    :   Add a step which marks the end of the most recently begun “if” or “while” block.

        |  |  |
        | --- | --- |
        | Returns: | the index of the step that was added |
        | Return type: | int |

    `getComputationStep`(*self*, *index*)¶
    :   Get the details of a computation step that has been added to the integration algorithm.

        |  |  |
        | --- | --- |
        | Parameters: | **index** (*int*) – the index of the computation step to get |
        | Returns: | - **type** (*ComputationType*) –   the type of computation this step performs - **variable** (*string*) –   the variable into which this step stores its result. If this step does not store a result in a variable, this will be an empty string. - **expression** (*string*) –   the expression this step evaluates. If this step does not evaluate an expression, this will be an empty string. |

    `getConstraintTolerance`(*self*) → double¶
    :   Get the distance tolerance within which constraints are maintained, as a fraction of the constrained distance.

    `getGlobalVariable`(*self*, *index*) → double¶
    :   Get the current value of a global variable.

        |  |  |
        | --- | --- |
        | Parameters: | **index** (*int*) – the index of the variable to get |
        | Returns: | the current value of the variable |
        | Return type: | double |

    `getGlobalVariableByName`(*self*, *name*) → double¶
    :   Get the current value of a global variable, specified by name.

        |  |  |
        | --- | --- |
        | Parameters: | **name** (*string*) – the name of the variable to get |
        | Returns: | the current value of the parameter |
        | Return type: | double |

    `getGlobalVariableName`(*self*, *index*) → std::string const &¶
    :   Get the name of a global variable.

        |  |  |
        | --- | --- |
        | Parameters: | **index** (*int*) – the index of the variable to get |
        | Returns: | the name of the variable |
        | Return type: | string |

    `getKineticEnergyExpression`(*self*) → std::string const &¶
    :   Get the expression to use for computing the kinetic energy. The expression is evaluated for every degree of freedom. Those values are then added together, and the sum is reported as the current kinetic energy.

    `getNumComputations`(*self*) → int¶
    :   Get the number of computation steps that have been added.

    `getNumGlobalVariables`(*self*) → int¶
    :   Get the number of global variables that have been defined.

    `getNumPerDofVariables`(*self*) → int¶
    :   Get the number of per-DOF variables that have been defined.

    `getPerDofVariable`(*self*, *index*)¶
    :   getPerDofVariable(self, index) -> PyObject \*

    `getPerDofVariableByName`(*self*, *name*)¶
    :   Get the value of a per-DOF variable, specified by name.

        |  |  |
        | --- | --- |
        | Parameters: | **name** (*string*) – the name of the variable to get |
        | Returns: | **values** – the values of the variable for all degrees of freedom are stored into this |
        | Return type: | vector< Vec3 > |

    `getPerDofVariableName`(*self*, *index*) → std::string const &¶
    :   Get the name of a per-DOF variable.

        |  |  |
        | --- | --- |
        | Parameters: | **index** (*int*) – the index of the variable to get |
        | Returns: | the name of the variable |
        | Return type: | string |

    `getRandomNumberSeed`(*self*) → int¶
    :   Get the random number seed. See setRandomNumberSeed() for details.

    `getStepSize`(*self*) → double¶
    :   Get the size of each time step, in picoseconds. If this integrator uses variable time steps, the size of the most recent step is returned.

        |  |  |
        | --- | --- |
        | Returns: | the step size, measured in ps |
        | Return type: | double |

    `setConstraintTolerance`(*self*, *tol*)¶
    :   Set the distance tolerance within which constraints are maintained, as a fraction of the constrained distance.

    `setGlobalVariable`(*self*, *index*, *value*)¶
    :   Set the value of a global variable.

        |  |  |
        | --- | --- |
        | Parameters: | - **index** (*int*) – the index of the variable to set - **value** (*double*) – the new value of the variable |

    `setGlobalVariableByName`(*self*, *name*, *value*)¶
    :   Set the value of a global variable, specified by name.

        |  |  |
        | --- | --- |
        | Parameters: | - **name** (*string*) – the name of the variable to set - **value** (*double*) – the new value of the variable |

    `setKineticEnergyExpression`(*self*, *expression*)¶
    :   Set the expression to use for computing the kinetic energy. The expression is evaluated for every degree of freedom. Those values are then added together, and the sum is reported as the current kinetic energy.

    `setPerDofVariable`(*self*, *index*, *values*)¶
    :   Set the value of a per-DOF variable.

        |  |  |
        | --- | --- |
        | Parameters: | - **index** (*int*) – the index of the variable to set - **values** (*vector< Vec3 >*) – the new values of the variable for all degrees of freedom |

    `setPerDofVariableByName`(*self*, *name*, *values*)¶
    :   Set the value of a per-DOF variable, specified by name.

        |  |  |
        | --- | --- |
        | Parameters: | - **name** (*string*) – the name of the variable to set - **values** (*vector< Vec3 >*) – the new values of the variable for all degrees of freedom |

    `setRandomNumberSeed`(*self*, *seed*)¶
    :   Set the random number seed. The precise meaning of this parameter is undefined, and is left up to each Platform to interpret in an appropriate way. It is guaranteed that if two simulations are run with different random number seeds, the sequence of random numbers will be different. On the other hand, no guarantees are made about the behavior of simulations that use the same seed. In particular, Platforms are permitted to use non-deterministic algorithms which produce different results on successive runs, even if those runs were initialized identically.

        If seed is set to 0 (which is the default value assigned), a unique seed is chosen when a Context is created from this Force. This is done to ensure that each Context receives unique random seeds without you needing to set them explicitly.

    `setStepSize`(*self*, *size*)¶
    :   Set the size of each time step, in picoseconds. If this integrator uses variable time steps, the effect of calling this method is undefined, and it may simply be ignored.

        |  |  |
        | --- | --- |
        | Parameters: | **size** (*double*) – the step size, measured in ps |

    `step`(*self*, *steps*)¶
    :   Advance a simulation through time by taking a series of time steps.

        |  |  |
        | --- | --- |
        | Parameters: | **steps** (*int*) – the number of time steps to take |

High performance molecular simulation on GPUs


### Navigation

- Application Layer
- Library Layer
  - Core Objects
  - Forces
  - Integrators
  - Extras

©2015, Stanford University and the Authors.
|
Powered by Sphinx 1.3.1
& Alabaster 0.7.7
|
Page source
